# Metabolic rewiring of the tumor microenvironment: therapeutic intervention of multi-pathway adaptations to impede cancer metastasis

**DOI:** 10.3389/fmolb.2026.1792455

**Published:** 2026-06-10

**Authors:** Kohhua Kaushik, Himanshu Singh, Kshitiz Jha, Rudra Pratap Singh, Navrinder Kaur

**Affiliations:** Amity Institute of Biotechnology, Amity University, Uttar Pradesh, India

**Keywords:** hypoxia, metabolic reprogramming, metastasis, tumor invasion, tumor microenvironment

## Abstract

Metabolic rewiring has an enormous impact on the tumor microenvironment (TME), which aids in proliferation of tumors and their metastasis This process involves alterations in cellular metabolism that satisfy the energetic and biosynthetic requirements of rapidly proliferating cancer cells, while simultaneously affecting the behavior of adjacent stromal and immune cells, thereby creating an environment conducive to tumor progression. Cancer cells exhibit increased glucose uptake and preferentially utilize glycolysis over oxidative phosphorylation for energy production, a phenomenon known as the Warburg effect. Moreover, changes in lipid metabolism yield vital elements for membrane production and energy storage, bolstering tumor growth and survival. Competition for metabolism and the build-up of immunosuppressive metabolites helps create an environment that fosters tumor progression and therapy resistance. The metabolic reprogramming of cancer cells affects TME by creating an environment devoid of nutrients and immune system suppression. Understanding the intricate metabolic relationships within the TME opens up possibilities for therapeutic approaches intended to disrupt these processes. A promising strategy to increase immune and stromal cells’ anti-tumor activity is to concentrate on modifying their metabolism. Additionally, resistance mechanisms in cancer treatment may be addressed by using metabolic inhibitors in addition to traditional therapies.

## Introduction

Cancer is a significant social, public health, and economic problem in the twenty-first century considering that it accounts for almost one in six death rates (16.8%) and one in four deaths (22.8%) from noncommunicable diseases (NCDs) worldwide. According to Seigel et al., the disease ranks among the top three causes of mortality in 177 out of 183 countries and is responsible for three out of ten premature deaths from NCDs globally (30.3% in individuals aged 30–69 years).

The lack of ability of cells to cope with stress and repair damage is the root cause of many cancers (Jelic et al., 2021). Despite a number of cancer treatment strategies, such as chemotherapy, radiation, immunotherapy, and surgery, patients with cancer cannot be completely cured (Zeng, 2018). Resistance to previous treatments is a major barrier towards effective treatment of cancer. Presently, developing highly effective anticancer therapies with few adverse effects is critically important.

New therapeutic targets can be identified by understanding the biological and metabolic processes of cancer. The metabolism of cancer cells is quite distinct from that of normal cells, and they are abnormal cells that are capable of rapid replication and regeneration. They are marked by constant migration, transformation, and proliferation and have the ability to eradicate healthy cells. Cancer cells employ special metabolic pathways to acquire molecules and energy to meet the needs of cell migration and proliferation considering their metabolism is much more active than that of normal cells. Numerous carcinogenic signalling pathways control the three main metabolic processes in cancer cells: glucose, amino acid, and lipid metabolism (Tang et al., 2021). Oncogene and tumor suppressor gene mutations interfere with intracellular signalling pathways, which have a direct impact on cancer cells’ metabolism. A mutated oncogene can start the metabolism of cancer cells right away. Under similar conditions, altered metabolic enzymes could propagate malignant transformation.

The alterations in cellular metabolism that enable cancer cells to meet the increased energy and biosynthetic requirements for rapid proliferation have been defined as metabolic rewiring in cancer. Tumor cells can adapt to various microenvironmental conditions and evade common regulatory mechanisms due to this phenomenon. A characteristic of this condition is that cancer cells favor glycolysis for energy production even in the presence of oxygen. This modification promotes proliferation by providing intermediates for biosynthetic pathways. These alterations in a number of metabolic pathways further promote long-term cell growth and aid in the development of tumors (Sharma et al., 2024).

Metabolic regulators in tumor cells have been identified by recent research as important factors influencing the complex reprogramming of tumor metabolism. This phenomenon creates an immunosuppressive tumor microenvironment, which interacts with immune cells, and regulates the tumor’s immune response. Understanding these regulators becomes essential for developing treatment approaches based on metabolism (Huang et al., 2025).

Tumor development, treatment response, and patient prognosis are all affected by metabolic variability in tumor cells. Distinct tumor locations experience various physiological adaptations as a result of genetic alterations, microenvironmental variables, and epigenetic modifications. For example, certain cancer cells might employ fatty acid oxidation or oxidative phosphorylation, whereas others may rely on glycolysis. In order to develop tailored therapies, it is essential to comprehend the tumor metabolic heterogeneity.

Recent research has demonstrated the impact of metabolic variability on tumor growth and invasion. Metabolic heterogeneity allows a minority of cells to cope with the challenging metabolic alterations required for proliferation, thereby facilitating tumor invasion and metastasis (Nong et al., 2023). During Epithelial-Mesenchymal Transition (EMT), cancerous cells leave the main tumor site, often changing from a highly glycolytic phenotype to the one that depends more on oxidative phosphorylation (OXPHOS) and non-glucose fuels such fatty acid oxidation (FAO). This demonstrates that metabolic reprogramming provides the energy and biochemical capacity necessary for migration, survival in the circulation, and eventual colonization of distant metastatic sites. Therefore, this intrinsic flexibility, fueled by a number of microenvironmental cues and genetic changes, allows a metabolically resilient subset of tumor cells to successfully complete the metastatic cascade.

Metabolic rewiring in cancer cells—alterations to their metabolic pathways that allow them to multiply and survive rapidly—has significant therapeutic implications. One intriguing approach to treating cancer is to target these metabolic changes. Inhibiting glycolysis, which is often elevated in cancer cells, is one such important strategy. The development of a network of metabolic regulators in the tumor microenvironment, their interactions with immune cells, and their impact on the tumor’s immune response were all covered in significant research studies. When this network is disturbed, an immunosuppressive environment emerges, weakening anticancer immunity (Huang et al., 2025).

According to Axe et al., another strategy focuses on amino acid metabolism alterations. It can be argued that a targeted approach to the amino acid pathways in question can selectively remove the cancer cells and leave normal cells untouched. This mechanism can be used by taking advantage of the unique metabolic requirements of cancer cells (Axe, 2023). Recent studies have emphasized the role played by metabolic reprogramming in supporting cancer metastasis. Mutations in the metabolic processes enable cancerous cells to live and proliferate to remote body parts. Understanding these metabolic changes is essential in order to come up with ways of preventing metastasis (Wei et al., 2020). Oncometabolism research focuses on the contribution of metabolic alterations of cancer cells in stimulating tumor growth and progression. Such changes often involve the accumulation of metabolites, also known as oncometabolites, which may disrupt the usual cellular functions and promote malignancy (Chen and Huang, 2024).

Indicatively, alterations in enzyme isocitrate dehydrogenase (IDH) lead to the appearance of an oncometabolite, D-2-hydroxyglutarate, which suppresses α-ketoglutarate-dependent dioxinases, affecting the regulation of epigenetics and gene expression (Liu et al., 2023). Recent studies have emphasized the role played by oncometabolites in various cancers. Chen et al. studied how the presence of metabolites such as succinate, fumarate, produced by cancerous and non-cancerous cells in the tumor microenvironment influenced the cell interactions and tumor development (Chen et al., 2022). Moreover, Liu et al. emphasized that oncometabolites become the distinctive components of cancer in humans: glioma, leukemia, neuroendocrine tumors, and renal cancer (Liu et al., 2023). A better understanding of oncometabolism is important in designing more specific therapies to disrupt such metabolic pathways, which may result in more effective therapies of cancer. The altered activity of IDH is a phenomenon of neomorphic enzyme function of mutant IDH1 (cytosolic) and IDH2 (mitochondrial). Wild-type IDH decarboxylates isocitrate oxidatively to produce α-ketoglutarate (α-KG) and NADPH. This normal reaction can be overridden by missense mutations at the active-site residues (R132 in IDH1, R140/R172 in IDH2) (Liu and Yang, 2021), which then allow a reductive conversion of α -KG to the oncometabolite D −2 -hydroxyglutarate (D −2 -HG) (Molenaar et al., 2018). The surplus D-2-HG is a competitive inhibitor of the 2-KG-dependent dioxygenases, reconfiguring the epigenetic and metabolic programs underlying tumorigenesis (Ma et al., 2015; Stein, 2016).

Recent research has brought attention to exploiting tumor cell metabolic characteristics to differentiate them from normal cells since uncontrolled infinite proliferation is a primary characteristic of malignancies (Martinez-Outschoorn et al., 2017; Pavlova et al., 2022). Rewiring of metabolism is an essential component of cancer biology, and the process of adaptive responses in tumor cells to the environment is enabled by it. Continued studies have shown the complexity of cancer metabolism; and have provided potential treatment intervention regimens. This review gives extensive information on the mechanism of alteration of various metabolic pathways; in addition to a comprehensive discussion on tumor-regulated mechanisms that can be used in combating tumor microenvironment related changes.

### Hypoxia (HIF-1 alpha) pathway mediated adaptation in TME and its therapeutic targeting

Hypoxia exhibits a reduction in oxygen levels, thereby causing significant changes in cell metabolism, primarily via hypoxia-inducible factor 1-alpha (HIF-1alpha) pathway. HIF-1 α is a protein that coordinates the process of cell adaptation to low oxygen, helping cells to survive and continue their functioning in hypoxic environments (Zhu et al., 2024). Under normoxic conditions, HIF-1 α is hydroxylated by the enzymes of prolyl hydroxylase domain (PHD), allowing ubiquitination and degradation degradation. HIF-1 α pathway is a key regulator of metabolic reprogramming in the hypoxic tumor microenvironment and supports the switch to aerobic glycolysis. This enables heightened survival of cancer cells (Chen et al., 2023). When activated by HIF-1 α, a group of essential glycolytic enzymes such as hexokinase and lactate dehydrogenase A (LDHA) become induced. Meanwhile, the key enzyme Pyruvate Dehydrogenase Kinase 1 (PDK1) is also activated, and this inhibits the movement of pyruvate into the TCA cycle.

**FIGURE 1:**
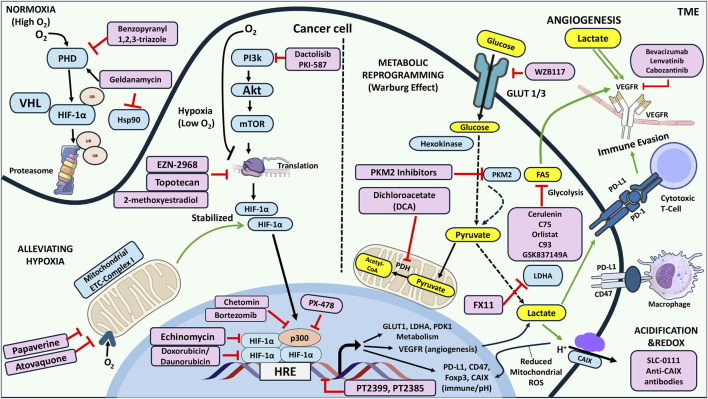
Hypoxia-driven metabolic reprogramming in the tumor microenvironment. HIF-1α stabilization during hypoxia facilitates glycolysis activating important enzymes (HK, LDHA) and suppressing the influx of pyruvate into the TCA. This conversion increases the production of lactate, angiogenesis (through VEGF), immune escape, and tumor survival. The therapeutic targets are HIF-1α inhibitors, PDK1 inhibitors and anti-angiogenic agents.

PDK1 prevents an important enzyme (PDH) that connects glycolysis with the mitochondria; thus, acting on pyruvate to convert it into lactate. In addition to the energy production, HIF-1 α activity is highly correlated with the maintenance of redox balance since the changed metabolic condition influences Reactive Oxygen Species (ROS) generation and results in a modification of mitochondrial activity to reduce ROS formation and inhibit apoptosis. Also, recent research serves as an indication of the therapeutic principles of targeting this axis, with PDK1 inhibition as an ability to reverse metabolic flux significantly influencing tumor survival and acting in conjunction with immunotherapy to enhance T-cell activity and alter the tumor microenvironment (TME) (Semenza, 2010) (Figure 1).

Therapeutic targeting of the HIF-1α pathway within the TME has attracted considerable attention as a method to hinder tumor progression and treatment resistance. Once stabilized, HIF-1α translocates into the nucleus and initiates transcription of genes required for immune evasion, cell survival, metabolism, and angiogenesis. In the hypoxic TME, HIF-1α promotes tumor growth by enhancing angiogenesis through VEGF overexpression, shifting cellular metabolism toward glycolysis, and promoting immune escape. For example, HIF-1α can induce the expression of the “do not eat me” signal CD47, impeding macrophage phagocytosis, and increase the expression of PD-L1 to suppress cytotoxic T cell activity.

A crucial strategy to reduce HIF-1α–driven tumor progression and therapy resistance is by abrogating its production or enhancing its degradation. Transcriptional inhibitors that disrupt HIF-1 activation via the coactivator p300 include Chetomin, which destabilizes the CH1 domain of p300 and impairs its interaction with HIF-1, and Bortezomib, which interferes with the HIF-1/p300 interaction (Karakashev and Reginato, 2015). Echinomycin, an antimicrobial cyclic peptide, binds directly to the HIF-1 recognition sequence, thereby impairing transcriptional activity (Karakashev and Reginato, 2015). The PI3K/Akt/mTOR pathway raises HIF-1 levels through increasing the HIF-1 protein translation rate; the translation inhibitors EZN-2968, Topotecan, and 2-methoxyestradiol downregulate HIF-1 by inhibiting its translation and nuclear translocation (Shirai et al., 2021). The molecular chaperone Hsp90 is crucial for HIF-1 stability, and pharmacologic inhibitors such as geldanamycin act by promoting HIF-1 ubiquitination and proteasomal degradation (Ikeda and Kakeya, 2021). PT2385 and PT2399 are potent, selective antagonists that directly bind and inhibit HIF-2α. More importantly, PX-478, an inhibitor of HIF-1α, suppresses the transcription and/or expression of Foxp3 and VEGF (Zaccagna et al., 2021). Benzopyranyl 1,2,3-triazole is an HIF-1 inhibitor which enhances HIF-1 hydroxylation and dose dependently downregulates VEGF expression (Albadari et al., 2019). The PI3K/AKT/mTOR axis significantly impacts the expression of HIF-1α, and its inhibition mitigates the levels of HIF-1α. Examples include Dactolisib, an agent that negatively modulates the mTOR/AKT/PI3K pathways (Peng et al., 2022), and dual inhibitors such as PKI-587, targeting the mTOR/AKT/PI3K signalling cascade and DNA damage repair pathway concurrently (Xie et al., 2021). Inhibition of HSP90, a factor involved in HIF-1 transcription, has shown therapeutic efficacy in bladder cancer (Yao et al., 2020).

Hypoxia and tumor metabolism can be addressed in a feasible manner in cancer treatment. The strategy is centered around crucial enzymes involved in metabolism. For instance, the GLUT1 inhibitor WZB117 targets glucose transporters GLUT1 and GLUT3 to inhibit the energy supply to the tumor (Pliszka and Szablewski, 2021). Inhibition of lactate dehydrogenase A by the potent and reversible competitive inhibitor FX11 and inhibition of PKM2 (pyruvate kinase M2) and glycolysis also occurs (Mohammad et al., 2019). Another important inhibitor of Pyruvate Dehydrogenase Kinase (PDK) and highly effective dichloroacetate triggers apoptosis in preclinical models by modulating the activity of PDH (Wang et al., 2021a).

Various inhibitors of Fatty Acid Synthase (FAS) like cerulenin, C75, orlistat, C93, and GSK837149A exist, and they display anticancer properties in cancer treatment (Du et al., 2022; Gouw et al., 2017). Given that hypoxia stimulates the process of angiogenesis, a treatment for it can be used to indirectly halt its progression. Inhibition of VEGF to its receptors by bevacizumab, a multi-kinase VEGF receptor inhibitor lenvatinib, and a tyrosine kinase cabozantinib constitute antiangiogenic therapy. Inhibition of certain molecular pathways also works well. The drug doxorubicin/daunorubicin inhibits the interaction between HIF-1 and the hypoxia responsive element (HRE) binding in the DNA (Abdullah et al., 2021). Inhibition of the mitochondrial electron transport chain, by virtue of which cells consume oxygen, may relieve hypoxia. The FDA-approved antispasmodic and its analogs, papaverine, can reduce the utility of oxygen by inhibiting Complex I of the electron transport chain (Singleton et al., 2021). Another inhibitor, atovaquone, in human serum albumin nanoparticles, has been effective in counteracting hypoxia (Wang, et al., 2021b).

Thus, in a nutshell, HIF-1α is regulated in response to changing oxygen levels and how that regulation feeds tumor progression. Under normoxia, abundant oxygen hydroxylates HIF-1α via PHD; this recruits VHL, targeting it for degradation by the ubiquitin pathway. Under hypoxia, low oxygen stabilizes HIF-1α, which then translocate into the nucleus, where it, along with p300, initiates transcription at the Hypoxia Response Element (HRE). Stabilized HIF-1α promotes metabolic reprogramming through increased expressions of GLUT1, LDHA, and PDK1; it supports angiogenesis via VEGFR and contributes to immune evasion by increasing PD-L1, CD47, and Foxp3. Paths like PI3K/Akt/mTOR increase the translation of HIF-1α and can be inhibited by therapeutic agents including but not limited to Dactolisib10. Other inhibitors include Chetomin, which prevents p300 binding, and PX-478 (Figure 1).

### Aerobic glycolysis and associated therapies

The tumor microenvironment (TME) is mainly driven by nutrient depletion and the accumulation of immunosuppressive substances. Additionally, immune suppression by T-cells results from deprivation in shared nutrients such as glucose. The metabolic byproducts contribute to shaping the tumor microenvironment. Immune suppression in the tumor microenvironment results from nutrient scarcity caused by high rates of glucose metabolism in cancerous cells. The tumor microenvironment is also conditioned by metabolic byproducts.

Lactate, particularly, is no longer just a waste product but a crucial signalling and energy substrate that has significantly altered the tumor microenvironment (TME) through a the “lactate shuttle” (Payen et al., 2020). In this mechanism, monocarboxylate transporters (MCTs) are used for lactate efflux from the glycolytic tumor cells, mostly MCT4, and influx into another tumor or stromal cell, mostly MCT1, where it acts as a strong substrate for energy metabolism. An important consequence is the reduction in the immunostimulatory effects of the acidic tumor microenvironment, which is a result of lactate accumulation. Therefore, targeting the two major lactate transporters, MCT1 and MCT4, provides a promising approach for preventing the supply of nutritional components to the tumor cells, reducing the immunosuppressive nature of the acidic tumor microenvironment, and improving the efficiency of current immunotherapy approaches for cancers. Lactate is in fact a *bona fide* signalling molecule, which binds the cell surface G protein coupled receptor GPR81 (also known as HCAR1) with physiological potency (Ishihara et al., 2022). Activation of GPR81 by lactate causes Gi protein signalling, decreases intracellular cAMP, and induces anti lipolytic, anti-glycolytic and glucose uptake in adipocyte, muscle and liver (Nordström et al., 2023; Rooney and Trayhurn, 2011).

On the other hand, mitochondria do not spend their lives idling or being dysfunctional. The adaptability of mitochondria leads to the progression of cancer, particularly metastases. Highly effective mitochondrial quality control (MQC) processes, such as mitophagy (Yiu et al., 2026), ensure that this adaptability—the ability to switch between glycolysis and OXPHOS—is maintained. Since OXPHOS can remain healthy due to the targeted mitochondrial degradation by mitophagy, the process of mitophagy has become a crucial component of metastases.

**FIGURE 2:**
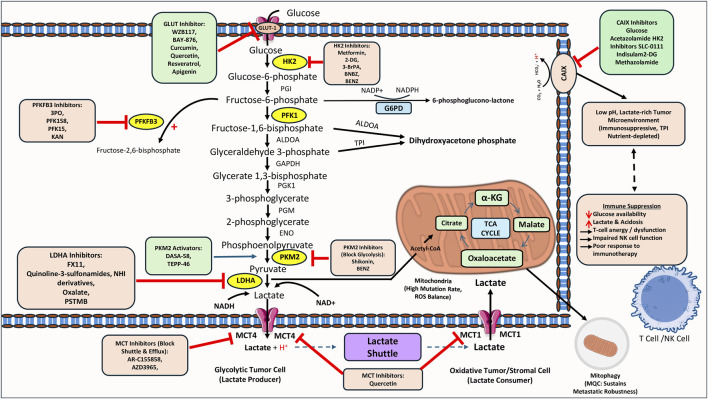
Aerobic glycolysis and metabolic plasticity in cancer cells. GLUTs, HK2, PKM2 and LDHA aid in the increased glucose uptake and lactate production (Warburg effect) in cancer cells. MTC1/4 lactate shuttling influences the tumor microenvironment. Mitochondria dynamics and mitophagy make sure that there is existing metabolic plasticity between OXPHOS and glycolysis.

The mitochondrial genome (mtDNA) is also important, to a great yet frequently underappreciated extent, within the pathogenesis of cancer cells (Copeland et al., 2002). Owing to the proximity to the electron transport chain in cells, the relatively leaky repair mechanism of mtDNA, combined with the higher mutation frequency due to the proximity to the electron transport chain in cells, mtDNA mutations are, therefore, a frequent occurrence within cancer cells. Yet, the role of the mitochondrial genome itself is becoming ever more important for the cancer pathogenesis, treatment, and drug response (Figure 2).

One of the most interesting approaches to cancer therapy aims at the tumor microenvironment’s glycolysis. The increased uptake of glucose and overproduction of lactate—is succeeded by an acidic and depleted environment. The metabolism turned upside down not only provides fuel for the high growth of cancer cells but also inhibits immune cells such as T cells and natural killer cells, supporting an immunosuppressive TME (Zhao J. et al., 2024).

Aerobic glycolysis provides carbon in glucose form to the oxidative pentose-phosphate pathway, where glucose-6-phosphate dehydrogenase (G6PD) generates NADPH that drives antioxidant systems (e.g., glutathione) and *de novo* lipogenesis (Lan et al., 2024). A fraction of the glycolytic intermediate 3 phosphoglycerate is diverted to the serine-glycine pathway through phosphoglycerate dehydrogenase (PHGDH) and produces serine and glycine to feed one-carbon (folate) metabolism to sustain nucleotide synthesis, methylation reactions and more NADPH production (Geeraerts et al., 2021). Therefore, the glycolytic shift maintains redox balance, membrane lipid biosynthesis and the one-carbon network that will support the growth of cancer cells.

To inhibit the proliferation of cancer cells due to the blocking of metabolism, enzyme-targeting strategies against aerobic glycolysis involve the inhibition of key enzymes and transporters. In the initial strategy, glucose transporters (GLUTs) are inhibited. Inhibitors such as the synthetic GLUT inhibitor WZB117 (De et al., 2023; Zhang et al., 2022; Temre et al., 2022) and the high-affinity GLUT1 inhibitor BAY-876 (Miller et al., 2024) reduce the proliferation of cancer cells by blocking glucose intake. Natural sources, including curcumin, quercetin, resveratrol, and apigenin, have been found to be effective GLUT inhibitors (Gunnink et al., 2016; Brito et al., 2016; Gwak et al., 2014; Wang et al., 2024a).

Hexokinase 2 (HK2) is another important target, as its overexpression has been observed in several types of cancer (Shan et al., 2022). Some well-known HK2 inhibitors are metformin, 2-deoxyglucose (2-DG), and 3-bromopyruvate (3-BrPA), although the last two are known to be toxic in dose-dependent manners (Bao et al., 2018; Pei et al., 2025). More selective HK2 inhibitors that target the glucose binding site and decrease glucose uptake are hydrazone derivatives such as Benitrobenrazide (BNBZ) and Benserazide (BENZ) (Zheng et al., 2021; Li et al., 2017).

Concerning the pyruvate metabolism pathway, there are activators and inhibitors for Pyruvate Kinase M2 (PKM2). The activator DASA-58 and TEPP-46 enhance the active tetramers of PKM2, thereby repressing cancer growth (Zhu et al., 2021; El-Far et al., 2022). By contrast, inhibitors that reduce PKM2 activity, including the natural compound Shikonin (Su et al., 2019; Liu et al., 2020; James et al., 2020), and BENZ (Dimitrijevs et al., 2023).

Stimulated hexokinase-2 (HK2) and activated phosphofructokinase-1 (PFK-1) drive the glycolytic flux towards high ATP production and supply a substantial number of glycolytic intermediates to anabolic pathways (Wang et al., 2025; Yuan et al., 2025). Moreover, elevated HK2 binds to mitochondria, linking glucose phosphorylation to anti-apoptotic signaling, and activating PFK-1 eliminates the primary rate limiting checkpoint, favoring proliferation and invasion (Yuan et al., 2025). In contrast, embryonic isoform PKM2 upregulation generates a kinetic bottleneck at the phosphoenolpyruvate-to-pyruvate path as the catalytic activity of PKM2 is reduced compared to PKM1/wild-type PK (Ishfaq et al., 2022). This sluggishness compels the upstream metabolites (e.g., 3 -phosphoglycerate) to be shunted onto side routes. Glucose 6-phosphate and 6-phosphogluconate are the glucose precursors in the oxidative PPP, which generates NADPH to support the antioxidant defense and lipid biosynthesis. The diverted 3-phosphoglycerate enters the PHGDH-mediated serine-glycine pathway, giving one-carbon units for nucleotide synthesis and further NADPH production via folate metabolism (Yi et al., 2012). As a result, the PKM2 bottleneck provides the cancer cells with metabolic plasticity: they maintain sufficient glycolytic ATP by channelling carbon into biosynthetic and redox-protective pathways, thereby boosting tumor growth, drug resistance and hypoxia tolerance (Ishfaq et al., 2022).

On the lactate side, Monocarboxylate Transporters (MCTs) and Lactate Dehydrogenase A (LDHA) are also targets. There are several types of LDHA inhibitors that can be categorised by their mechansim, including oxalate (An et al., 2017; Valvona and Fillmore, 2018; Altinoz and Ozpinar, 2022) and 1-(phenylseleno)-4-(trifluoromethyl) benzene named PSTMB (Kim et al., 2019), NADH cofactor-competitive compounds such as FX11 and quinoline-3-sulfonamides (Li et al., 2023; Rellinger et al., 2017; Liu et al., 2022; Hou et al., 2021; Oshima et al., 2020; Woodford et al., 2020), and dual-competitive compounds such as derivatives of the N-hydroxyindole compound, NHI (Xing et al., 2023; Angulo-Elizari et al., 2023). Moreover, there are non-specific therapeutics such as quercetin for MCTs, strong and selective inhibitors such as AR-C155858 and its orally accessible counterpart AZD3965 are being developed to prevent lactate efflux (Nancolas et al., 2015; Guan et al., 2018; Beloueche-Babari et al., 2020).

To summarize, Glucose is transported into the cell via the GLUT1 transporters and funnelled into glycolysis, resulting in Pyruvate. Instead of transported into mitochondria for oxidative phosphorylation, pyruvate is converted to Lactate via LDHA and then pumped out of the cell via MCT4 into the surrounding environment (Li and Cui, 2023). The transport of lactate out of the cancer cell acidifies the extracellular space and produces an acidic tumor microenvironment. This acidification is considered to be another highly important cause, together with nutrient starvation, of T-cell anergy and impaired T-cell function. Inhibition of key points such as GLUT transporters (e.g., WZB117, BAY- 876), HK2 (e.g., Metformin, 2-DG), PKM2 (inhibitors like Shikonin or activator TEPP-46), and MCTs (e.g., AZD3965) will disrupt the lactate shuttle and efflux (Figure 2).

In conclusion, focusing on glycolysis within the TME and altering the tumor mechanism offers a versatile approach to cancer treatment. However, more research and clinical development is essential to completely utilize glycolysis-targeted methods in oncology.

### Effect of glutamine in TME and its inhibition

Within the tumor microenvironment, there is a process of reprogramming of metabolism where there is diversion of the metabolism of glutamine, which directly affects tumor growth and the immune response. Tumor cells preferentially utilize glutamine for their high rate of division and survival, thereby drawing it from the surrounding environment and reducing its availability for the immune cells, which directly affects their function (Hensley et al., 2013; Recouvreux et al., 2024).

This is induced through the upregulation of glutamine transporters, for example, SLC1A5, and enzymes glutaminase (GLS), with the induction of oncogenes c-Myc to promote this process through the induction of glutamine uptake and metabolism. In addition, this modification assists the evasion of the immune response through the induction of a starvation condition unfavorable to tumor-infiltrating lymphocytes (Yoo et al., 2020).

**FIGURE 3:**
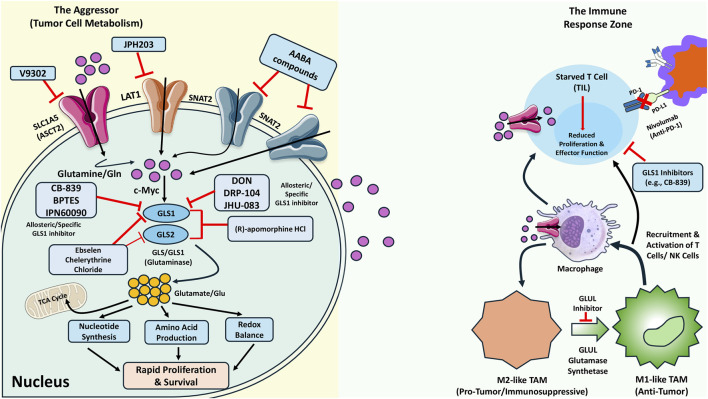
Glutamine metabolism and immune modulation in the tumor microenvironment. The tumor cells promote glutamine intake through transporters (SLC1A5) and breakdown through GLS, which promotes the proliferation and redox balance. Immunologic suppression by glutamine depletion and glutamine metabolism through inhibitors of GLS (e.g., transporter inhibitors) increases anti-tumor immunity.

Metabolic reprogramming is also a factor in the modulation of tumor-associated macrophages (TAMs). Glutamine metabolism is central to the IL-4-mediated M2 modulation of TAMs, which are thought to mediate immunosuppression and tumor advancement. The inhibition of glutamine synthetase (GLUL) helps induce an M1-like state in TAMs, which augments their ability to attract T cells, thus improving anti-tumor immunity (Liu et al., 2017) (Figure 3).

The approach of targeting glutamine metabolism within the tumor microenvironment presents an attractive modulation strategy for therapy. Glutamine is crucial for the facilitation of the TCA cycle, maintaining the redox state, as well as providing substrates for the generation of nucleotides and amino acids. “Glutamine addiction” has often been described within cancer cells that highly depend on glutamine for supporting their high growth rate to stay alive. In addition to promoting tumor growth, this metabolic reprogramming produces a competitive environment that may hinder immune cell function within the TME (Wise and Thompson, 2010; Ziki and Colnot, 2024).

Glutaminase (GLS) is also a key enzyme for the cellular processing of glutamine, converting it into glutamate for the TCA cycle. Inhibitors for this enzyme, termed GLS1, include CB-839 or telaglenastat, with positive trial results achieved for both lab and human studies (Hartwick and Curthoys, 2012; 81- Shukla et al., 2012). CB-839 has been shown to increase the potency of immune checkpoint therapy, called nivolumab, for a variety of cancers, these include renal cell carcinoma, non-small cell lung carcinoma, and melanoma. Another prototype is DRP-104 or sirpiglenastat, which is the prodrug form of the all-spectrum glutamine antagonist DON. It is designed as an approach with lower systemic toxicity and is primarily activated within the tumor microenvironment (Recouvreux et al., 2024).

Novel strategies to interfere with glutamine metabolism are being studied apart from direct enzyme inhibition. V9302 is a competitive inhibitor of the glutamine transporter and binds selectively to SLC1A5 to inhibit glutamine entry into cancer cells (Schulte et al., 2018). In the case of breast cancer, V9302 may induce autophagy to modulate reactive oxygen species accumulation. In conjunction with conventional chemotherapeutic drugs or anti-PD-1 treatment, V9302 demonstrates increased anti-cancer activity in terms of reduced tumor growth, reversed chemoresistance, and heightened immune reactions (Szemerédi et al., 2024; Li et al., 2022).

Inhibitors of the glutamine pathway often interact with glutamine transport or the enzyme GLS itself. AABA analogs are examples of glutamine transport inhibitors. They prevent the transport of glutamine even when cells overexpress SNAT2 to compensate for the lack of ASCT2. JPH203 (Okunushi et al., 2020) is an anticancer agent that inhibits the transport of the branched-chain amino acid by binding to the L-type amino acid transporter 1 (LAT1), which stimulates the uptake of amino acids.

Inhibition by an older drug, DON (6-Diazo-5-oxo-norleucine), is due to interference with a variety of glutamine-dependent enzymes, involving a two-step mechanism-based inhibition manner (Investigational Drug Branch CTTP, 1979; Lemberg et al., 2018; Pinkus, 1977). In gliomas, GLS1 inhibitors, such as BPTES (Seltzer et al., 2010; Elgadi et al., 1999), have been found to have a pronounced action in cell proliferation; BPTES is a strong uncompetitive inhibitor of the GLS1 isoform.

Ebselen, chelerythrine chloride, and (R)-apomorphine hydrochloride are additional glutaminase inhibitors identified by screening. While (R)-apomorphine hydrochloride exhibits comparable efficacy against both isoforms, ebselen and chelerythrine chloride are substantially more effective against GLS1 than GLS2. The latter two are competitive inhibitors, whereas ebselen acts by non-competitive inhibition, potentially by covalent adduct formation (Thomas et al., 2013; Sakurai et al., 2006; De La Barre et al., 2011; Santos and Schulze, 2012).

Tumor cells become addicted to glutamine across distinct zones of the TME. The mechanism of entry for glutamine into the cell is through transporters, including but not limited to SLC1A5, also known as ASCT2. Glutaminase, or GLS/GLS1, then catalyses glutamine into Glutamate. Glutamate feeds the TCA cycle, supporting nucleotide synthesis, production of other amino acids, and redox balance, maintaining the requirements of rapid cell growth. The illustration identifies Zone-1 as the site of aggressor tumor metabolism, Zone-2 depicted as the extracellular TME, and Zone- 3, the site of an immune response at which glutamine has become scarce, leading to starved T cells and the presence of M2-like tumor-associated macrophages characterized by their pro-tumor feature. Principal approaches include the use of GLS1 inhibitors, such as CB-839 and BPTES, and SLC1A5 inhibitors, such as V9302, to starve the tumor from critical sources of nitrogen and carbon (Figure 3).

Replenishment of food to immune cells can enhance their anticancer activity by preventing glutamine engagement or metabolism in cancer cells. In addition, immunotherapies including immune checkpoint inhibition can be used in combination with the glutamine metabolism inhibitors to optimize the therapeutic outcomes (Dong C et al., 2024). Overall, this concept of direct prevention of tumor growth, through modulating the immunological niche in the TME, and intervention on the glutamine metabolism provides a complete method of treating cancer. Studies and clinical trials on the potential of glutamine-targeted medicines are continuing, and it is hoped that they will be implemented as part of comprehensive treatment regimens of cancer.

### Lipid metabolism alterations in tumor cells and its inhibition

Metabolic rewiring in the tumor microenvironment (TME) is an important alteration in lipid metabolism that supports progression of the tumor and the expression of immune responses. In order to meet the demands of their rapid multiplication and to survive under such conditions as the absence of oxygen and the deficit of nutrients, tumor cells increase lipid intake, synthesis, and oxidation. Such a metabolic adaptation influences the behaviour of stromal and immune cells in its immediate environment, promotes tumor expansion and contributes to the establishment of an immunosuppressive environment (Xu et al., 2021; Jin et al., 2023).

**FIGURE 4:**
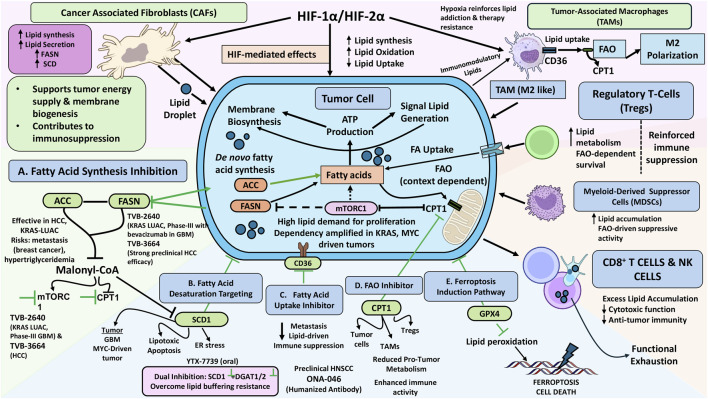
Lipid Metabolism and Therapeutic Targeting in the Tumor Microenvironment. Tumor and stromal cells promote lipid synthesis, uptake, and oxidation to boost growth and survival. The most important regulators are FASN, ACC, SCD1 and CD36. Disturbed lipid metabolism facilitates immunosuppression and tumor-growth, which are targets of therapy.

An increase in lipid synthesis and secretion characterizes lipid metabolic re-programming in cancer-associated fibroblasts (CAFs). CAFs are indispensable parts of the TME. They increase the activity of enzymes like fatty acid synthase (FASN) and stearoyl-CoA desaturase (SCD) leading to an increment of lipids production that could be utilized by tumor cells as energy and biosynthesis of membranes. Lipids produced by CAFs alter immune homeostasis and also promotes an immunosuppressive microenvironment that contributes to tumor progression (Jin et al., 2023) (Figure 4).

In the TME, immune cells also experience lipid metabolism. A case in point is the tumor-associated macrophages (TAMs) that increase lipid uptake and fatty acid oxidation (FAO), a process that contributes to their polarization into an M2-like pro-tumor phenotype. Regulatory T cells (Tregs) and myeloid-derived suppressor cells (MDSCs) show similar effects which facilitate lipid metabolism, thereby reinforcing their immunosuppressive functions. The initial, rate-limiting, stage of tryptophan (Trp) degradation is performed by indoleamine-2,3-dioxygenase 1 (IDO1) and its paralogue, indoleamine-2,3-dioxygenase 2 (IDO2), which convert tryptophan change into N-formyl-kynurenine and then into kynurenine (Kyn) (Liu et al., 2018). Extracellular Trp, which stimulates the amino -acid -sensing kinase GCN2 and suppresses mTOR signalling in infiltrating T cells, triggers cell cycle arrest, anergy and apoptosis in the tumour microenvironment (TME) via depleting high IDO1 expression (Opitz et al., 2020). The accumulated Kyn ligand occupies the aryl-hydrocarbon receptor (AHR) on dendritic cells, myeloid-derived suppressor cells (MDSCs) and naive CD4 + T cells, re-programming them to tolerogenic phenotypes and boosting regulatory T cells (Tregs) (Platten et al., 2015). The joint action of Trp depletion and Kyn -AHR signalling forms an immunosuppressive niche that inhibits the activity of effector CD8 + T -cells and facilitates tumour immune evasion (Triplett et al., 2018). Conversely, the CD8^+^ T cells and the natural killer (NK) cells may accumulate excess lipids that may negatively affect their effector activities and their anti-tumor capabilities (Jin et al., 2023). The therapeutic manipulation of the lipid metabolism within the tumor microenvironment (TME) is an interesting way of treating cancer. Tumor cells often have their lipid metabolism changed so as to evade immunity, grow rapidly, and survive stress. Examples of this reprogramming of the metabolism include increased synthesis of *de novo* fatty acids, better lipid uptake and changes in lipid storage and oxidation pathways. Inhibiting lipid metabolism in cancer involves inhibiting key enzymes that participate in fatty acid absorption, desaturation, and synthesis and pathway products including ferroptosis. One of the major targets is fatty acid production (FAS), and the key control points are Acetyl- CoA carboxylase (ACC) and Fatty acid synthase (FASN). ACC inhibitors are very active in preclinical models of hepatocellular carcinoma (HCC) (An and Li, 2025) and oncogenic KRAS-driven lung adenocarcinoma (LUAC); nonetheless, their preclinical progress is hampered by complications, including unexpected hypertriglyceridemia in humans and progressive metastasis in cancer of breast.

Inhibition of FASN has been more promising; TVB-2640 has progressed to clinical trials in oncogenic KRAS-mediated LUAC and currently is in a Phase III trial in relationship with bevacizumab in glioblastoma multiforme (GBM) (100) (Kelly et al., 2023). Moreover, TVB-3664 has already shown the active anticancer properties in preclinical models of HCC (Wang et al., 2022; Che et al., 2017; Ntellas and Chau, 2024). Besides reducing the synthesis of fatty acids, FASN inhibitors also cause malonyl-CoA to accumulate, which inhibits carnitine palmitoyltransferase 1 (CPT1) on the mitochondria and functions as a conserved endogenous inhibitor of the protein complex mTORC1.

The enzyme promoting lipid desaturation, Stearoyl-CoA Desaturase 1 (SCD1) happens to be another significant therapeutic target. Inhibiting SCD1 results in lipotoxic apoptosis by encouraging the buildup of saturated fatty acids and endoplasmic reticulum stress. It is effectively characterized in GBM and MYC-driven malignancies. One clinical-stage oral SCD1 inhibitor with excellent blood-brain barrier penetrance, YTX-7739, showed high efficacy in GBM rodent models, although none of SCD1 inhibitors have been transferred to clinical trials addressing cancer (Eyme et al., 2023). This is because co-inhibiting DGAT1/2 is plausible to increase SCD1 inhibition in lipids tumors which become resistant through lipid buffering.

The third way is to inhibit the consumption of fatty acids and the transmembrane protein, CD36. Anti-CD36 antibodies are found to prevent the metastasis in preclinical models of squamous cell carcinoma (HNSCC) (Pascual et al., 2017). ONA-046, a humanised CD-36 inhibitory antibody, is presently being developed as a treatment (Dolgin, 2023). Besides, within the cell death signalling platform, ferroptosis, an iron-dependent method of non-apoptotic cell death can be preclinically provoked by disrupting intrinsic antioxidant pathways including glutathione peroxidase 4 (GPX4) (Doll et al., 2019; Pope and Dixon, 2023; Li et al., 2024; Freitas et al., 2024; Bersuker et al., 2019).

Lipid metabolism in the tumor microenvironment is influenced by conditions of hypoxia and acidosis, which consequently alter the way fats are synthesized, utilized, and transported. In hypoxic regions of the tumor, HIFs promote the synthesis and uptake of lipids and reduce the oxidation of lipids for energy production. Targeting such hypoxia-mediated pathways may provide new avenues for the treatment of cancer (Munir and Zaidi, 2024).

Lipid metabolism is at the core of tumor expansion and the immunosuppressive milieu present within the tumor microenvironment (TME). Cancer cells have a huge lipid requirement for their rapid growth, which involves the synthesis of cell membranes, ATP production through fatty acid oxidation (FAO), and signalling lipid production. This high lipid demand is more evident in KRAS and MYC-driven cancers. Under low oxygen availability, the signalling of HIF-1α/HIF-2α takes this further, promoting lipid synthesis (FASN and SCD), lipid uptake (CD36), and the synthesis of lipid droplets. The diagram further identifies the role of lipid metabolism and accumulation in regulating the function of immune cells. The diagram encourages tumor-associated macrophages (TAMs) to be pro-tumorigenic M2 macrophages, supports the survival of regulatory T cells (Tregs), and stimulates the growth of suppressive myeloid-derived suppressor cells (MDSCs) through FAO. Simultaneously, it inhibits the killing ability of CD8^+^ T cells and NK cells. Lastly, it identifies the areas of potential therapeutic targeting: inhibiting fatty acid synthesis (e.g., FASN inhibitors such as TVB-2640 deployed in glioblastoma (Kelly et al., 2023) and KRAS-driven lung cancers), inhibiting Fatty Acid uptake (CD36 inhibitors such as ONA-046), and inhibiting FAO, as well as inducing ferroptotic cell death via GPX4 inhibition (Bartolacci et al., 2022). It seeks to bring down lipid-mediated immune suppression and augment anti-tumor immunity (Figure 4).

Apart from feeding growth, the lipid metabolism is emerging as a modulator of the immune landscape in the TME. Once lipid processes go out of equilibrium, possible accumulation of various immunosuppressive lipid species can dampen the actions of immune cells like macrophages and T cells. Modulation of lipid metabolism would be able to strengthen anti-tumor immunity and improve responses elicited by immunotherapies.

The overall dual punch of hitting the lipid metabolism in the TME is that it directly can stave off tumor growth and reshape the immune environment to support treatment. Researchers remain on the lookout for drugs that target lipids with a view to weaving them into broader, more effective strategies for treatment.

### Immunotherapeutic strategies targeting the TME

The tumor microenvironment (TME) is effectively a very complex ecosystem that plays a significant role in modulating immune cells such as T-cells and Macrophages based on tumor metabolism. Tumor-Associated Macrophages (TAM) are effectively very diverse immune cells; further, the modulation or differentiation of these TAMs is highly dependent on tumor metabolism within the TME. TAMs are effectively polarized to be mainly M2-type and predominantly pro-tumorigenic, which is supported physiologically by high levels of Oxidative Phosphorylation (OXPHOS) and high levels of Fatty Acid Oxidation (FAO), despite having a formal M1/M2 classification.

The immune suppression caused by TME is largely due to a severe instance of metabolite co-occurrence as a direct function of its metabolite specializations. Suppressing anti-tumor immune responses is heavily dependent on a single strategy, as employed by TAMs and stromal cells in particular, even when thoroughly leveraging any available nutrient. For example, in some instances of the tumor microenvironment, TAMs become the primary glucose consumers in place of effector T-cells, primarily due to the massive utilization of glycolysis by the effector T-cells that fail to utilize glucose, a nutrient that is basically needed for the growth of effector T-cells.

The tumor-derived pathways involving NF-kappaB and STAT3 that upregulate metabolically active pathway components involved in OXPHOS and FAO, in addition to an M2 phenotype, often drive this conversion. It is both mandatory to hamper the tumor-friendly microenvironment and to resurrect an antitumor response in T lymphocytes to target these pathways of dependence on glucose or fatty acids in particular for the immunosuppressive tumor-associated macrophages (Wang et al., 2024b).

With activated effector T-cells (Teffs), especially cytotoxic CD8^+^ cells, essentially depending on a high rate of glycolysis to supply the required amount of ATP and biosynthetic intermediates, anti-tumor T-cells need a strong and dynamic metabolic program (Brest and Luciano, 2021). Tumor cells and stromal components, on the other hand, fiercely compete with one another for essential resources, particularly glucose and amino acids, making the Tumor Microenvironment (TME) extremely nutrient-scarce. T-cell dysfunction and anergy are primarily caused by this nutritional deficiency because the T-cells lack the ability to maintain the high glycolytic flux necessary for efficient cytotoxicity.

**FIGURE 5:**
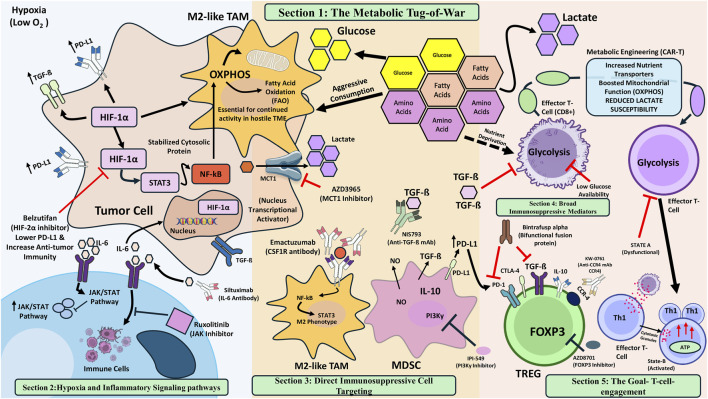
Immunotherapeutic Approaches for Modulating Metabolic and Signalling Pathways in Tumor Microenvironment (TME). Metabolic competition and immunosuppressive metabolites disable T-cell activity and induce macrophage polarization towards tumors. The therapeutic approaches focus on the immune checkpoints, cytokine receptor, and metabolic pathways to replenish anti-tumor immunity.

Immunotherapy includes a lot of emphasis on improving the metabolic fitness of therapeutic T-cells, like in CAR-T cell therapy, in order to overcome these TME-imposed restrictions. In addition, to achieve better and longer-lasting anti-tumor responses, metabolic engineering techniques such as increasing nutrient transporter expression for improved competition, boosting mitochondrial function to favor OXPHOS and sustain function in low-glucose conditions, and altering T-cells to be less susceptible to inhibitory factors like lactate are used (Gu et al., 2025) (Figure 5).

Similarly, promoting the metabolic fitness of T-cells, for example, through an enhancement of glycolytic activity or an inhibition of tumor metabolite-mediated suppression, could also make immunotherapy more effective (Scharping and Delgoffe, 2021). Interventions could inhibit tumor-supportive metabolism and make therapeutic responses better through modulation in immune cell metabolism in TME (Lim and Rathmell, 2020).

Therapeutic targeting of immunological pathways in the TME has, therefore, been an important part of modern cancer therapy to counter immune suppression and enhance antitumor immunity. The tumor microenvironment is composed of immune cells such as T cells, DCs, macrophages, and regulatory T cells. These cells show a dynamic interaction with the tumor cells that influences the nature of disease progression and the extent of treatment efficacy.

Hypoxia-related pathways are among the primary targets, given the fact that the low oxygen TME is known to inhibit CTLs and promote the infiltration of Tregs. The signalling cascade of HIF-1 alpha/STAT3/NF-B is blocked by drugs such as Belzutifan, a HIF-2 alpha inhibitor that decreases the expression level of PD-L1 and decreases immunosuppression (Choueiri et al., 2021; Jonasch et al., 2021; Choueiri et al., 2023). The signalling cascade induced by the acidic environment produced by the upregulation of lactate produced by AZD3965, a Monocarboxylate Transporter 1 Inhibitor, negatively affects the function of the CD8^+^ T cells, which upregulate the inhibitory receptor proteins, such as PD-1, CTLA-4, on the surface of T cells (Halford et al., 2023).

Activation of the PI3K/Akt-CREB pathway by Lactate -activated GPR81 stimulates secretion of the pro-angiogenic factor amphiregulin and induces the formation of new blood-vessels in breast tumours (Lee et al., 2016). GPR81-lactate interaction in dendritic cells reduces intracellular cAMP, inhibits the expression of MHC II on the surface and the presentation of antigens, which leads to a less immunogenic tumour microenvironment (Brown et al., 2020). PD-L1 is also upregulated by GPR81 signalling: lactate-GPR81 activation decreases cAMP/PKA activity, liberating the transcriptional co-activator TAZ, which along with TEAD promotes PD-L1 transcription in tumour cells (Feng et al., 2017) and the loss of GPR81 decreases the level of PD-L1 in the same (Chen et al., 2021). The combination of these mechanisms promotes immune evasion in gastric cancer by lowering antigen presentation, increasing PD-L1, and attracting regulatory T cells through CX3CL1 induction (Su et al., 2024) as well as altering the immune landscape, as evidenced by a reduction of CD8+/FOXP3+ T-cell ratios in GPR81-high breast cancers (Li et al., 2023).

It is also essential to target inflammatory cytokine signalling, particularly the IL-6 family of cytokines, which stimulate the JAK/STAT pathway to support the growth and survival of tumor cells. By increasing Th1 and CD8^+^ T effector cells and decreasing Th17 differentiation, blocking IL-6 signalling with antibodies like Siltuximab (van Rhee et al., 2014; Brighton et al., 2019) or JAK inhibitors like Ruxolitinib (Moskowitz et al., 2021; Dao et al., 2020; Landen et al., 2024) can improve immunotherapy.

SLC6A14 is an amino-acid symporter that is broad-specific and highly upregulated in many cancers (e.g., colorectal, breast, AML) and imports most proteinogenic amino acids to drive tumor growth via mTOR and JAK2/STAT3 signaling; knock-down inhibits proliferation and migration (Lu et al., 2022; Mao et al., 2021). This carrier depletes the extracellular amino acids in the tumour microenvironment, indirectly lowering immune cells nutrients, and contributing to T-cell dysfunction in (Dejure et al., 2020). SLC43A2 is a high-affinity methionine transporter that is over-expressed in tumour cells to compete with the CD8^+^ T lymphocytes for methionine. This T-cell methionine deficit reduces intracellular S -adenosyl -methionine, inhibits H3K79me2 and STAT5 production, and causes T -cell exhaustion (Bian et al., 2020). Pharmacological or genetic suppression of tumor SLC43A2 restores the levels of methionine, histone methylation and enhances anti-tumor immunity (Bian et al., 2020). SLC6A14 and SLC43A2 work together to change the availability of nutrients in the tumour microenvironment, promoting cancer cell metabolism and decreasing immune cell activity.

Direct targeting of immunosuppressive cell populations is another important approach. Agents like IPI-549 (PI3K-gamma inhibitor) are used to target Myeloid-Derived Suppressor Cells (MDSCs), which suppress T-lymphocytes through factors like NO, TGF-β, and IL-10 and upregulate PD-L1 (Hong et al., 2023). Emactuzumab (CSF1R antibody) inhibits receptors essential for polarization and activity of tumor-associated macrophages (TAMs), which are primarily of the immunosuppressive M2 phenotype (Gomez-Roca et al., 2022).

Anti-CCR4 antibodies (KW-0761) (130- Fujikawa et al., 2023) or FOXP3 inhibitors (AZD8701) (Revenko et al., 2022) are used to target regulatory T-cells (Tregs), which are significant inhibitors of CTLs via surface markers such CTLA-4 and PD-1 and production of TGF-β and IL-10. Additionally, a significant focus is on the broad immunosuppressive cytokine Transforming Growth Factor Beta (TGF-β), which inhibits Th1 and Th2 differentiation and lowers T cell cytotoxicity. Treatments range from monoclonal antibodies like NIS793 to bifunctional fusion proteins like Bintrafusp alfa (targeting TGF-β and PD-L1) (Bauer et al., 2023; Rajan et al., 2024; Yoo et al., 2023).

There is an existing interaction between tumor cells, immune cells, and the metabolic environment presented by the TME and indicates how the veil of immunosuppression can be lifted through therapeutic approaches. It divides the interaction into five strategic areas: Zone 1: The Metabolic Battle—tumor cells rapidly utilize nutrients, leaving T-effectors malnourished. Zone 2: Signalling by hypoxia mediated by HIF-1 alpha and pro-inflammatory pathways (JAK/STAT), which may be treated with medications like Belzutifan and Ruxol. Zone 3: Immunosuppressive cell targeting (M2-like TAMs, MDSCs, Tregs) with direct approaches such as Emactuzumab and IPI. Zone 4: Immunosuppressive cytokine mediators, such as TGF-β and PD-L1. Zone 5: The endgame—that is, re-engaging the T cells, switching them from a shutdown ‘State A’ to a turned-on, ‘State B’ metabolically active state through metabolic re-programming and checkpoint modulation (Figure 5).

### ECM-TME interaction and its destabilization to improve therapy

The Extracellular Matrix (ECM) is increasingly recognized as a dynamic component of the TME, actively shaping cancer cell metabolism and immune evasion in addition to its conventional role as a structural scaffold (Dzobo and Dandara, 2023). The ECM’s distinct stiffness and composition, which are frequently determined by Cancer-Associated Fibroblasts (CAFs), function as powerful biophysical cues that can directly influence the metabolic state of tumor cells. For example, they can determine the cell’s overall sensitivity to nutrient availability and its dependence on glucose. The extracellular matrix may also serve as a source of nutrients and also a regulator of catabolism. New literature indicates that the extracellular matrix (ECM) has been actively involved in the metabolism of specific amino acids in cancer cells, such as by enhancing the breakdown of tyrosine in cases of local/systemic nutrition deficit (Nazemi et al., 2024).

In addition, the TME is also an effective immunoregulator by the ECM (Lu and Chen, 2024). By controlling immune cell recruiting, differentiating and activating, together with the regulation of TAM and T-cell infiltrations, the extracellular matrix remodeling not only physically prevents immune cell infiltration, but also establishes biochemical conditions that contribute to the creation of an immunosuppressive microenvironment. The engagement between the immune system and the extracellular matrix is one of the most important obstacles to the success of immunotherapy.

The metabolic strategy of a cancer cell is established together with the biophysical and biochemical properties of the extracellular matrix (ECM). Specifically, the unique stiffness and composition of the ECM actively control the reliance of the cell on glucose, where signals generated by the matrix have higher relevance than the simple availability of nutrients (Henke and Naba, 2020; Guerrero-López et al., 2025). Individual cell types living in a hard matrix environment (often abundant in specific extracellular matrix (ECM) constituents, such as collagen) may be more reliant on glucose uptake and glycolysis, but the same cells living in a softer matrix may be more prone to alternative energy sources.

Such mechano-metabolic connection suggests that the physical organization of the TME is what defines how sensitive the cell is to nutrient availability, which points to the necessity of treatment strategies, which consider the physical context of the surrounding extracellular matrix.

ECM targeting in the microenvironment of tumor is another promising strategy that can be used in cancer treatment. The ECM is a protein-polysaccharide net besides offering structural support to tissues needed in growing, spreading of cancer cells, and resistance to therapy. One of the principal constituents of the ECM of the tumor environment is caused by the deposition and cross-linking of collagen and other ECM proteins, raising stiffness (Mai et al., 2024). This complicates the infiltration of the immune cells into the environment and the penetration of drugs beyond the cancerous cells in addition to increasing the entry of invading cancerous cells. This has been demonstrated to increase the immune efficacy by reducing its stiffness by means of immune cell infiltration to deliver effective immunotherapy against cancer cells (Feng et al., 2024).

One of the key contributors to changes in the ECM within malignancies is cancer-associated fibroblasts or CAFs (Mai et al., 2024). In order to facilitate tumor growth and meeostasis, these fibroblasts secrete enzymes as well as constituents of the ECM that result in changes to the matrix. One such therapeutic target would be to target CAFs or the function of CAFs in modifying the ECM. Therapeutic targets that will aim to inhibit changes to stiffness in the ECM, as well as halt growth of tumor parenchymal cells, include enzymes that facilitate collagen cross-linking, such as lysyl oxidase or LOX. Decreasing the strength of the extracellular matrix through inhibition of LOX will inhibit cell migration.

**FIGURE 6:**
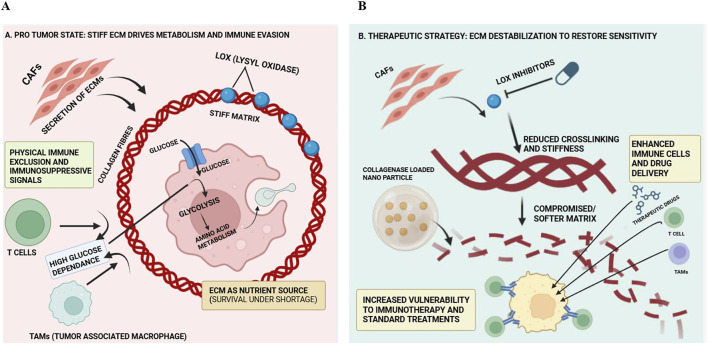
Mechanisms of ECM-Mediated Immune Evasion and Therapeutic Sensitization in tumors. **(A)** The ECM stiffness and composition have an impact on cancer cell metabolism and nutrient dependency. **(B)** The immune infiltration and delivery of drugs improve with targeting of ECM remodeling (e.g., LOX inhibition, collagen degradation) to improve therapeutic response.

The tumor microenvironment can also progress from a resistant one to a one that we can control by treatment, via targeting the extracellular matrix (ECM). In the Pro-Tumor Environment, CAFs secrete ECM proteins, which are cross-linked via the enzyme LOX to form a stiff and dense matrix. Stiffness is a barrier, encourages immune evasion, and promotes a highly glycolytic and high glucose-using tumor metabolism, with the ECM being a nutritional resource for tumor cell survival (Figure 6A). However, when it comes to targeting this pro-tumor environment, a Therapeutic Strategy is proposed, which uses LOX inhibitors and collagenase-loaded nanoparticles to degrade those fibres, rendering a soft and weakened ECM, which improves drug penetration and immune cell infiltration, so rendering the tumor much more susceptible to therapies (Figure 6B).

Extracellular matrix also can be a physical barrier; unlimited access of therapeutic agents including immune cells and nanoparticles is highly restricted. One method that is being studied is where nanoparticles are loaded with collagenase to break down or modify the structure of the extracellular matrix thereby enhancing the delivery and efficacy of drugs. Targeting of the TME ECM is a multi-modal approach to treating cancer through interfering with tumor supporting structures, stimulating immunity and transportation of therapeutic drugs in the tumor. Combining ECM-targeted medicines with current therapies is still being studied with the consideration of the fact that they can ultimately increase the results of cancer patients.

## Integration of metabolic networks in tumor systems

Tumor metabolism is no longer considered as a group of discrete biochemical pathways but a multi-layered network which encompasses intracellular metabolism, intercellular interactions and systemic host physiology. Integration of metabolic networks is the dynamic interaction of core metabolic pathways (glycolysis, oxidative phosphorylation (OXPHOS), lipid metabolism, and amino acid metabolism) and regulatory circuits mediated by oncogenic signaling, epigenetic alterations, and the tumor microenvironment (TME). This systems-level perspective has become crucial for comprehending tumor progression, metabolic plasticity, and therapeutic resistance.

Cancer metabolism is associated at the cellular level with large-scale rewiring of interconnected metabolic pathways. Classical observations like the Warburg effect, preferential aerobic glycolysis, are just a node in a larger metabolic network which encompasses mitochondrial respiration, glutaminolysis, and lipid biosynthesis (Tufail et al., 2024). These pathways do not occur in isolation, instead, they are closely intertwined with each other via the presence of common intermediates, redox balance, and energy needs. An example is that glycolytic intermediates are used in the pentose phosphate pathway leading to the production of nucleotides and tricarboxylic acid (TCA) cycle intermediates are used in the synthesis of lipids and amino acids (Al-Horani, 2026). The inter-relationship between these cells allows the cancer cells to sustain biosynthetic fluxes even as they adjust to varying nutrient and oxygen supply.

Another trait of tumor metabolic networks is metabolic plasticity, which is the ability of cancer cells to change pathways in response to changes in their environment. Instead of depending solely on glycolysis or OXPHOS, tumor cells have hybrid phenotypes, modulating the fluxes through the pathways to maximize survival and growth. Oncogenes (e.g., MYC, PI3K/AKT/mTOR) and tumor suppressors (e.g., p53, LKB1) mediate this flexibility by being key players in connecting signaling networks to metabolic pathways (An et al., 2017). These regulation centers combine nutrient sensing, energy status, and stress signals, thus coordinating the global metabolic responses throughout the network.

In addition to intracellular integration, intercellular metabolic crosstalk in the tumor microenvironment has a profound influence on tumor metabolism. Through communication of metabolites (lactate, amino acids and lipids) between cancer cells and stromal cells, immune cells, fibroblasts and endothelial cells, cancer cells engage metabolically with these cells. As an example, glycolytic tumor cells may release lactate, which can be utilized by oxidative tumor cells or stromal elements, which can create a symbiosis of metabolism. The TME also fine-tunes the activity of immune cells based on competition for nutrients and signaling from metabolites. This links metabolism to immune evasion and how effectively a treatment works (Chapman and Chi, 2024). Recent work highlights that these interactions form a distributed metabolic network that spans across several cell types and is not just limited to cancer cells.

Notably, tumor metabolic networks go as far as the host organism level, where the host metabolism is combined with tumor growth. Tumors may change the entire metabolism of the body through induction of cachexia, changes in nutrient availability and reprogramming of liver and muscle metabolism. In contrast, host factors such as diet, microbiome composition, and endocrine signaling can influence tumor metabolic states. Recent 2025 review points out that tumor-host metabolic interactions inform the so-called tumor macroenvironment, which highlights that cancer metabolism should be conceptualized as a systemic process and not an entirely cell-autonomous process. This systemic integration highlights the relevance of investigating cancer metabolism *in vivo*, wherein organism-level influences play a role in metabolic heterogeneity (Altea-Manzano et al., 2025).

Spatiotemporal heterogeneity of tumors is another important aspect of metabolic network integration. Oxygen, nutrient and pH gradients form unique metabolic niches between tumor regions. Poorly perfused areas prefer glycolysis and reductive metabolism whereas better-perfused areas may be more dependent on mitochondrial respiration. Due to these spatial differences, there are heterogeneous metabolic conditions that live together in the same tumor, which leads to resistance to therapy and disease development. There is also an increased complexity of temporal dynamics as the metabolic states change as tumours progress, metastase, and respond to treatment. An example is that metastatic cells reprogram their metabolism to fit their new organ niches, which indicates the plasticity and context-dependentness of tumor metabolic networks (Peng-Winkler and Fendt, 2026; Dong and Li, 2026).

Embarkation of metabolic networks also is closely connected with epigenetic and signaling networks and is embedded in a multilayered regulatory system. The chromatin-modifying enzymes are cofactor-dependent on metabolites like acetyl-CoA, α-ketoglutarate, and S-adenosylmethionine, which connects metabolic conditions to gene expression. This two-way communication forms feedback loops whereby epigenetic landscapes are altered by metabolic changes, and so regulate the expression of metabolic enzymes. This type of integration enables tumors to rapidly alter their metabolic pathways in response to environmental or therapeutic stressors. The advancement of systems biology and computational modeling has been instrumental in elucidating the intricacies of tumor metabolic networks. Genome-scale metabolic models (GEMs), flux balance analysis (FBA), and multiscale modeling methods can be used to utilize omics data (genomics, transcriptomics, metabolomics) to make predictions. The models have the ability to model metabolic flux distributions, discover key pathways and make predictions of responses to metabolic interventions. Recent research emphasizes the significance of multiscale models that combine intracellular metabolism with tissue-level and whole-organism processes, making it possible to create patient-specific digital twins to be used in precision oncology. These methods are important in converting the knowledge of metabolic networks into strategies that can be implemented in clinical practice (Vanoni et al., 2024).

The combined nature of the tumor metabolism has both challenges and opportunities as far as therapeutic treatment is concerned. When a single metabolic pathway is targeted, other metabolic pathways tend to be compensatory activated. Thus, network-based targeting can be an effective approach to the therapeutic strategies, either by the combination of several pathway-inhibitors or by the simultaneous perturbation of metabolic and signaling networks. Also, the ability to disrupt tumor-promoting metabolic ecosystems by targeting metabolic interactions in the TME, like lactate transport or amino acid exchange, is promising. New therapies are also seeking to take advantage of metabolic weak points generated by mutations in oncogenes or nutrient addictions.

To sum up the analysis, the emergence of metabolic networks in tumors represents a highly dynamic, adaptive, hierarchical system, which functions at the various biological scales. Intracellular pathway rewiring is not the only way to regulate tumor metabolism, but also intercellular communication, local environmental pressures, and systemic interactions with the host. Knowledge of this interconnected network architecture is the key to deciphering tumor biology and coming up with more effective treatment plans. In the future, studies that combine high-resolution multi-omics data with systems-level modeling will further clarify the underlying principles of organizing metabolic networks and develop new intervention opportunities in treating cancer.

## Limitations and translational challenges in metabolic rewiring in cancer

Despite the fact that the notion of metabolic rewiring has made significant strides in improving our knowledge of tumor biology, there are still a number of key limitations, existing controversies, and barriers to translation into clinical practice. The predominant restriction is the extensive use of *in vitro* experimental models, especially, immortalized cancer cell lines, which are cultivated under non-physiological nutrient and oxygen conditions. Such models do not model the complexity of the tumor microenvironment (TME), such as the presence of hypoxia, nutrient gradients, and stromal-immune interactions. As a result, metabolic addictions that have been found *in vitro* might not be relevant to tumor behavior *in vivo*. In fact, even though aerobic glycolysis is still a diagnostic of cancer metabolism, growing evidence indicates that tumors still have much oxidative phosphorylation (OXPHOS) potential, underscoring the shortcomings of simplified metabolic models (Punnasseril et al., 2025).

The other significant limitation is the dominance of the static and reductionist approaches. A lot of studies are based on endpoint measurements of abundances of metabolites or gene expression, which give a picture of a very dynamic metabolic network at only one point in time. Tumor metabolism is also flux-based and adaptive in nature; thus, the absence of longitudinal and flux-based analyses limits the determination of actual metabolic weaknesses. Moreover, there is a tendency to underrepresent metabolic heterogeneity, both intertumoral and intratumoral. Cancerous stem cell tumor subpopulations, such as cancer stem cells, have different metabolic phenotypes and can dynamically alternate between glycolysis and OXPHOS in response to environmental or therapeutic stress, thus complicating therapeutic targeting (Keoh et al., 2025).

The area is also marked by a number of controversial issues that are still unresolved. The Warburg effect as a universal metabolic characteristic of cancer has been a longstanding focus, which is increasingly disputed. Recent findings indicate that metabolic plasticity as opposed to a fixed glycolytic phenotype is a hallmark of tumors. It is common to find cancer cells in mixed metabolic states and may reorganize their metabolic responses in context, invalidating the argument of targeting a particular pathway, like glycolysis. This has also cast doubt on the soundness of the metabolic addiction hypothesis because tumors tend to counteract a blockage of pathways by network redundancy and alternative use of substrates (Khan et al., 2025).

The other controversial subject is the nature of metabolic interactions in the TME. Although other studies suggest a cooperative relationship between two metabolic symbiosis (e.g., lactate exchange between hypoxic and oxidative tumor cells), others focus on competitive interactions, especially between tumor and immune cells over a necessary nutrient. Recent studies emphasize that the TME is a complex and context-specific metabolic ecosystem, in which cooperation and competition coexist and are influenced by environmental limitations and cellular composition (Djelti et al., 2026).

These conflicting opinions illustrate the necessity of spatially and temporally resolved analyses in order to fully understand metabolic crosstalk. There are significant challenges in translating metabolic rewiring to clinical applications. One of the barriers is the relative lack of specificity of the metabolic inhibitors because most of the targeted pathways play a vital role in the normal functioning of cells, leading to a small therapeutic index. Moreover, metabolic plasticity allows fast adaptation to therapeutic pressure which is an aspect of drug resistance. Metabolic reprogramming has been found to be a key mechanism that leads to resistance to chemotherapy, targeted therapy and immunotherapy, whereby tumor cells re-organize metabolic fluxes to maintain survival in stress (El-Sehrawy et al., 2025). As an example, tumors that are resistant to therapy tend to migrate towards mitochondrial metabolism, indicating the importance of combinatorial and adaptive therapeutic approaches (Qiu et al., 2025).

The other major obstacle is the absence of strong and clinically viable biomarkers. In contrast to genomic changes, metabolic states are very dynamic and respond to systemic factors like diet, microbiome, and host metabolism, and are hard to standardize in clinical practice. Despite new opportunities of metabolomics and multi-omics integration, technical and analytical limitations are still present to translate into clinical practice. Moreover, the design of clinical trials of metabolic therapies is in development. Numerous trials on early stage have shown little effectiveness, in part because of poor stratification of patients and lack of knowledge of tumor metabolic conditions. Recent reviews have highlighted that combinations of metabolic inhibitors with immunotherapy or targeted therapies could be effective, but these ideas would demand more specific mechanistic understanding and patient selection based on biomarkers (Khan et al., 2025).

Finally, although metabolic rewiring is a key biomarker of cancer, its practical implementation is limited by the experimentation ability, lack of conceptual clarity, and therapeutic considerations. To solve these problems, physiologically relevant models will have to be created, dynamic and spatial metabolic studies will be integrated, and systems-level approaches will be embraced. The next-generation studies are advised to focus on establishing context-specific metabolic weaknesses and the development of rational combination therapy to defeat resistance and enhance clinical outcomes.

## Discussion

In case of tumor invasion and metastasis, metabolic rewiring is necessary because it enables the cancer cells to adapt to the tumor microenvironment and continue with the metastatic process. These metabolic changes are instigated by unregulated oncogenic programs like PI3K/AKT, which are found in breast cancer, in which site-specific metabolic patterns influence metastases (Drapela and Gomes, 2021; Li et al., 2023b; Bergers and Fendt, 2021).

Epithelial-to-mesenchymal transition (EMT) that enhances plasticity and invasiveness of cancer cells is closely linked with metabolic reprogramming. The activity of mitochondria and reactive oxygen species (ROS) increase to promote EMT, and oncometabolites, including fumarate, promote epigenetic modification to enhance metastatic potential (Martínez-Reyes and Chandel, 2021; Li et al., 2023b; Elia and Haigis, 2021). Also, the metabolism of fatty acids (FA) plays a role in this process since the metastatic cells increase the activity of fatty acid carriers like CD36 to meet the energy needs of dissemination (Falletta et al., 2022; Bergers and Fendt, 2021).

Tumor and stromal cells metabolism also contribute to metastasis by redistribution of nutrients and formation of immunosuppressive microenvironment. To support the production of energy and redox balance, metastatic cells treat the amino acids obtained by the stroma, like glutamine, in the organs, primarily the brain and liver (Wang et al., 2023; Bergers and Fendt, 2021). This is based on the hypothesis of the seed and soil which refers to the selectivity of metastatic sites towards cancer cells that have a metabolic advantage (Wang et al., 2023).

Metabolic markers have the capability of predicting the metastasis with mitochondrial ROS related with greater metastatic potential and bone metastases-related changed serine metabolism (Elia and Haigis, 2021; Li et al., 2023b). Exosomal integrins may also be used to predict organ-specific metastasis by remodelling pre-metastatic niches (Wang et al., 2023). Targeting metabolism-related weaknesses such as the uptake of FA and metabolism of amino acids can offer potentially effective treatment alternatives to prevent metastasis (Ganguly and Kimmelman, 2023; Krieg et al., 2024). Understanding the metabolic rewiring, EMT regulation and stromal interactions is vital to improve cancer therapy directed at metastasis (Drapela and Gomes, 2021; Martínez-Reyes and Chandel, 2021).

The metabolic vulnerabilities have a promising area of endeavour where they can prevent metastases. Blockage of the FA uptake pathways, or glutamine metabolism, may disrupt some of the energy supply lines important to metastatic cells. Indicatively, inhibition of metastatic seeding by CD36 analogues have been denoted to be effective in preclinical models. Besides, treatments intended to decrease the levels of ROS or alter mitochondrial metabolism might dampen the process of EMT and metastatic properties.

In conclusion, metabolic reprogramming is instrumental in all phases of metastasis, including EMT and spreading as well as during colonization of foreign organs. The comprehension of the interaction of oncogenic signalling and cellular metabolism and the contribution of the stromal niche in the development of therapeutic approaches that preferentially inhibit metastatic progression is of significant value. Hence, due to increasing research in this field, it is possible to tackle the metabolic amenities of metastasizing cells and help decrease the mortality attributed to cancer in a number of ways.
